# Targeting a heterologous protein to multiple plant organelles via rationally designed 5′ mRNA tags

**DOI:** 10.1186/1754-1611-7-20

**Published:** 2013-09-08

**Authors:** Mathias J Voges, Pamela A Silver, Jeffrey C Way, Matthew D Mattozzi

**Affiliations:** 1 Wyss Institute for Biologically Inspired Engineering, Harvard University, Boston, MA 02115, USA; 2 Department of Systems Biology, Harvard Medical School, Boston, MA 02115, USA; 3 Chimerion Biotechnology Inc, Cambridge, MA 02138, USA; 4 Department of Biotechnology, Technische Universiteit Delft, 2628 BC, Delft, Netherlands

**Keywords:** Protein targeting, Alternative splicing, Signal embedding, Localization, Peroxisome, Chloroplast, Cytosol, Organelle, *Nicotiana benthamiana*

## Abstract

**Background:**

Plant bioengineers require simple genetic devices for predictable localization of heterologous proteins to multiple subcellular compartments.

**Results:**

We designed novel hybrid signal sequences for multiple-compartment localization and characterize their function when fused to GFP in *Nicotiana benthamiana* leaf tissue. TriTag-1 and TriTag-2 use alternative splicing to generate differentially localized GFP isoforms, localizing it to the chloroplasts, peroxisomes and cytosol. TriTag-1 shows a bias for targeting the chloroplast envelope while TriTag-2 preferentially targets the peroxisomes. TriTag-3 embeds a conserved peroxisomal targeting signal within a chloroplast transit peptide, directing GFP to the chloroplasts and peroxisomes.

**Conclusions:**

Our novel signal sequences can reduce the number of cloning steps and the amount of genetic material required to target a heterologous protein to multiple locations in plant cells. This work harnesses alternative splicing and signal embedding for engineering plants to express multi-functional proteins from single genetic constructs.

## Background

Plant cells harbor many distinct compartments that share some overlapping function, or are functionally associated in metabolic pathways and development. To enable complex metabolic engineering, plant engineers will require tools to direct single transgenes to multiple compartments. For example, re-engineering photorespiration [1,2] and isoprenoid synthesis [3,4] will involve both the chloroplasts and peroxisomes.

A number of synthetic N-terminal and C-terminal extensions are readily available to target heterologous proteins to desired subcellular compartments, such as the chloroplast, peroxisome, mitochondrion, endoplasmic reticulum or the nucleus. Issues around protein targeting have arisen in (1) studying protein function in a coordinated fashion [5,6], (2) improving holistic plant metabolic engineering efforts [7-9] and (3) increasing yields attained by molecular farming and other protein factory applications [10]. One approach to target proteins to more than one location involves cloning multiple genetic copies, each containing a different localization peptide. Each copy must be introduced by successive retransformation, or alternatively, by backcrossing single transforms [11]. These procedures are time-intensive and yield transformants with multiple spatially distinct copies of a protein expression cassette. Coordinate expression may not be ensured due to context-dependent regulatory effects and/or homology-based silencing [12]. Although dual targeting to certain organelles may instead be achieved by adding a second localization peptide [10], this approach is limited to the possible combinations that can be made from available N- and C-terminal extensions.

Here we describe a simple technique for targeting of transgenic proteins to multiple organelles, specifically the chloroplast, peroxisome, and cytosol. This combination of organelles is particularly interesting due to their close functional association in photorespiration, isoprenoid biosynthesis, β-oxidation and other metabolic processes [3,13,14].

## Results

### Design for multiple-compartment localization by alternative splicing: TriTag-1 and TriTag-2

To construct TriTag-1 and TriTag-2, a chloroplast-targeting region (CTPa) was taken from protein-L-isoaspartate methyltransferase (*PIMT2*, At5g50240). *PIMT2* is a ubiquitous repair protein, converting exposed isoaspartate residues to aspartate or asparagine residues in aging polypeptides [15,16]. Various mRNAs produced from *PIMT2* are produced by alternative transcription initiation sites and alternative splicing events [16]. In nature, different isoforms are often produced from an individual gene, via the exclusion or inclusion of coding sequences from its mRNA by alternative splicing [17-19]. The spliceforms produced from the 3′ transcription initiation site target the protein to the chloroplast when the targeting sequence is retained, and to the cytosol when it is not.

A peroxisome targeting sequence, PTS2, containing the RLx_5_HL nonapeptide [20], was taken from the transthyretin-like S-allantoin synthase gene (*TTL*; At5g58220). This synthase catalyzes two steps in the allantoin biosynthesis pathway [21]*.* At least two spliceforms are produced from *TTL* from internal alternative acceptor junctions. The translated proteins are targeted to the peroxisome if they retain the internal PTS2 site and to the cytosol if the site is removed [21].

**Figure 1:**
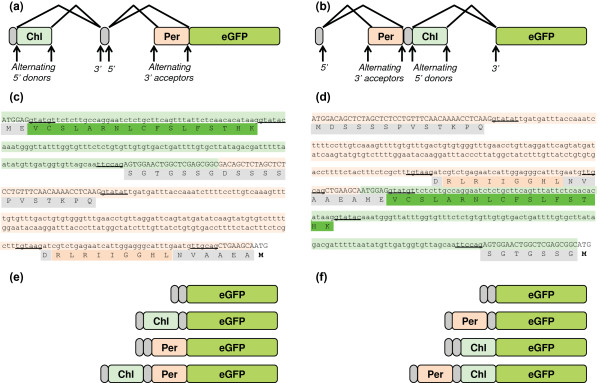
**Design of TriTag-1 and TriTag-2, with alternatively spliced chloroplast-targeting *PIMT2 *and peroxisome-targeting *TTL *tags. (a, b)** Splice diagrams of TriTag-1 **(a)** and TriTag-2 **(b)**, showing non-targeting sequences (gray), chloroplast targeting sequences (light green), peroxisome targeting sequences (tan), and the enhanced GFP coding sequence used in transient expression experiments. **(c, d)** Design of TriTag-1 **(c)** and TriTag-2 **(d)** sequences. The ATG codon at the end corresponds to the first residue of the GFP open reading frame. Alternatively spliced targeting regions are highlighted. Donor and acceptor dimers are underlined. The light green DNA sequences derive from the *PIMT2* 5′ coding region [16] and include sequences required for chloroplast targeting (green). The light tan DNA sequences derive from the *TTL* 5′ coding region [21] and include sequences encoding the peroxisome targeting sequence (tan). **(e, f)** Final mRNAs species resulting from alternative splicing of TriTag-1 **(e)** and TriTag-2 **(f)**.

Harnessing the sequences attained from the above genes, we designed two novel 5′ mRNA tags (TriTag-1 and TriTag-2) that targeted the translated GFP to chloroplast, peroxisome and/or cytosol using alternative splicing (Figure 1). An initial pre-mRNA comprising of the entire gene is initially transcribed. This pre-mRNA is then alternatively spliced. The terms “donor” and “acceptor” sites refer to the 5′ (GT) and 3′ (AG) splicing junctions, respectively. For example, in Figure 1a, two 5′ donor junctions flank an mRNA sequence that encodes a chloroplast-targeting tag. The resultant protein may or may not exhibit a chloroplast-targeting tag, depending on whether the encoding pre-mRNA was excised as an intron.

TriTag-1 contains the elements in this order: a short sequence of *PIMT2* containing the start codon, two alternative donor sites flanking chloroplast transit peptide CTPa, a single acceptor site, a short exon that encodes glycine and serine residues, a single donor site, and two alternative acceptor sites flanking the peroxisome targeting sequence PTS2 of the *TTL* gene (Figure 1a,c). In TriTag-2 the positions of the sequences taken from genes *PIMT2* and *TTL* are reversed (Figure 1b,d). Both tags are designed so that the two alternative splicing events occur independently of each other. As a result, mRNAs encoding chloroplast, peroxisomal, and cytoplasmically localized proteins are expected (Figure 1e,f).

### Design for dual-targeting by signal embedding: TriTag-3

**Figure 2:**
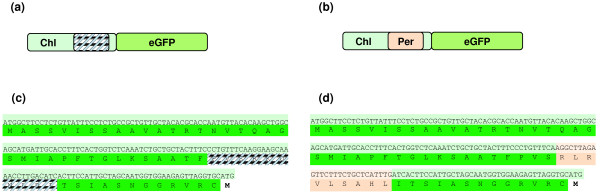
**Design of TriTag-3, containing peroxisome-targeting *TTL *tag embedded within the chloroplast-targeting *rbcS1 *tag. (a, b)** Diagrams of potato Rubisco *rbcS1* chloroplast targeting tag CTPb **(a)** and TriTag-3 **(b)**, showing chloroplast targeting sequences (light green), peroxisome targeting sequences (tan), flexible regions (diagonals), and the enhanced GFP coding sequence used in transient expression experiments. **(c, d)** CTPb **(c)** and TriTag-3 **(d)** sequences. The ATG codon at the end corresponds to the first residue of the GFP open reading frame. The light green DNA sequences derive from the *rbc*S1 5′ coding region [1] and encode a chloroplast targeting sequence (green). The light tan DNA sequences **(d)** code for a consensus PTS2 signal (tan). The PTS2 sequence is embedded within a flexible region of CTPb (green diagonals).

For targeting to two intracellular locations with a single N-terminal extension, we embedded a peroxisome targeting sequence within a chloroplast targeting sequence (TriTag-3, Figure 2b,d). The 9-aa *TTL* peroxisome-targeting peptide PTS2 [15,16] was placed within the chloroplast targeting region from the ribulose-1,5-biphosphate carboxylase (RuBisCO) small-subunit *rbc*S1 (CTPb, Figure 2a,c, GenBank: X69759.1) [22], substituting for a poorly conserved segment in the CTP that is predicted to form an unfolded segment (determined by the PROFbval tool on the ROSTLAB server [23]). Specifically, the amino acids closest to the N-terminus of the protein are the most effective at differentiating between targeting to the chloroplast and the mitochondria. Inspection of the *A. thaliana* chloroplast-targeted proteins revealed a decrease in conservation of CTPs toward the C-terminus [24,25]. Based on these findings, we placed PTS2 at the 40th amino acid. The resulting targeting peptide, TriTag-3, retains a predicted structure similar to the native CTPb in terms of flexibility. We determined that proteins containing the N-terminal TriTag-3 extension would be targeted to the peroxisomes and chloroplasts using TargetP [26] and PeroxisomeDB 2.0 [27].

### Subcellular localization of GFP controls in transient assays

**Figure 3:**
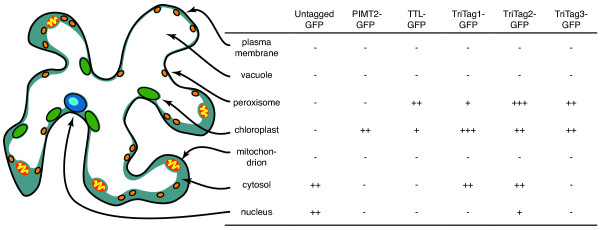
**Compartments of a typical tobacco leaf epidermal cell.** Here we illustrate their relative sizes and locations within the cell, and the relative expression levels observed via confocal microscopy.

We tested the targeting properties of the TriTag-GFP fusions in *Nicotiana benthamiana* leaf epidermal cells using biolistic particle delivery (Bio-Rad Helios Gene Gun) for transient expression. This method allows for rapid transient expression of GFP in a few scattered cells per leaf. This is ideal for observing GFP expression *in vivo* via fluorescence in a single cell in isolation [17,28]. Expression was controlled by the P_ENTCUP2_ constitutive promoter and the nopaline synthetase (NOS) termination signal [29]. Images were taken by confocal microscopy (Leica SP5 X MP, Buffalo Grove, IL 60089 United States) 48–96 hours after particle delivery. The subcellular fluorescent localization patterns in transfected leaf tissue were compared to chlorophyll autofluorescence; untagged GFP, and constructs designed for expression in the chloroplasts and peroxisomes as controls. A diagram summarizing a typical tobacco leaf epidermal cell and observed expression patterns is provided (Figure 3).

**Figure 4:**
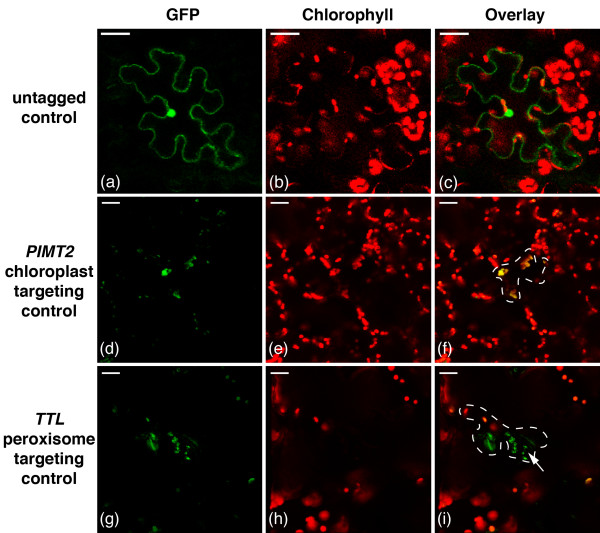
**Typical transient expression pattern controls in *N. benthamiana *leaf epidermal cells.** GFP channel **(a, d, g)**, chlorophyll autofluorescence **(b, e, h)** and composite **(c, f, i)** images were generated using confocal microscopy. Comparison with a,b and c allow for identification of non-targeted GFP by a pattern of cytosolic, plasma membrane and nuclear expression. The PIMT2-GFP fusion in d, e and f shows expression in the chloroplast. The TTL-GFP fusion in **g**, **h**, and **i** shows expression throughout the cell but some preference for small organelles we believe to be peroxisomes (arrow). Scale bar is 25 μm.

Untagged GFP expressed in the cell periphery and a single large organelle we presumed to be the nucleus. GFP expression in a single organelle per cell that was larger than a chloroplast and not co-localized to a chloroplast was presumed to be in the nucleus. As the vacuole takes up 90% of the cell volume (Figure 3), expression in the cell periphery is likely cytosolic (Figure 4a,b,c; see also [30]). GFP fused to the chloroplast targeting peptide of *PIMT2*[15,16] showed expression in chloroplasts (Figure 4d,e,f). GFP fused to the peroxisome targeting peptide of *TTL*[20,21] (slightly modified from [21] by the addition of a start codon) showed expression in organelles that resemble peroxisomes (Figure 4g,h,i). The *TTL*-tagged GFP was sometimes localized to a subset of the chloroplasts in the transfected cells, but much weaker than was observed using the TriTag constructs (below).

### Subcellular localization of TriTag-1 and TriTag-2 fused GFP

**Figure 5:**
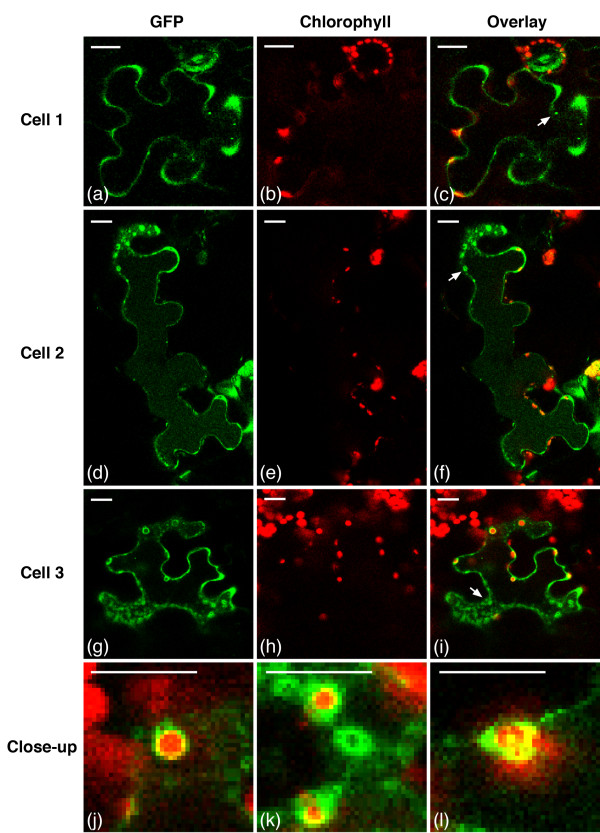
**TriTag-1-GFP distribution in *N. benthamiana *epidermal leaf cells.** Images of GFP expression **(a, d, g)**, chlorophyll autofluorescence **(b, e, h)**, and composite **(c, f, i)** of three different cells were generated by confocal microscopy. The arrows in **(c, f, i)** indicate an organelle believed to be a peroxisome. **(j-k)** Close-up of chloroplasts from **(i)**. **(l)** Close-up of a chloroplast from **(f)**. Scale bars 25 μm.

**Figure 6:**
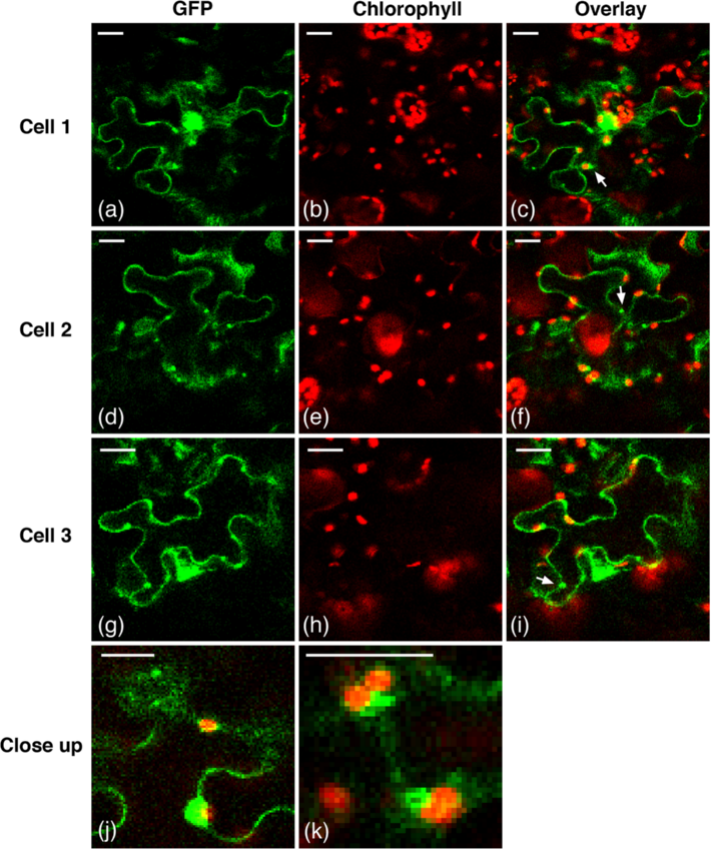
**TriTag-2-GFP distribution in *N. benthamiana *epidermal leaf cells.** Images of GFP expression **(a, d, g)**, chlorophyll autofluorescence **(b, e, h)**, and composite **(c, f, i)** of three different cells were generated by confocal microscopy. The top arrow in **(i)** indicates a chloroplast from which a low GFP signal is observed; the (bottom) arrow in **(f, i)** indicates a GFP-fluorescing peroxisome. **(j-k)** Close-up of chloroplasts and peroxisomes from **(f, i)** respectively. Scale bars 25 μm.

TriTag-1 and TriTag-2 were designed to target the resultant protein to either the chloroplast or peroxisome via alternative splicing of the encoding mRNA. The two tags contain the same elements but in complimentary orders (Figure 1). Both tags showed localization to the cytoplasm, chloroplast, and peroxisome (Figures 5 and 6). GFP expression (Figures 5a,d,g and 6a,d,g) was compared to the autofluorescence of chloroplasts (Figures 5b,e,h and 6b,e,h) and the size and distribution pattern of peroxisomes (Figure 4g,h,i). Transient expression of TriTag-1-GFP resulted in cytosolic and chloroplast localization, with the latter inferred by chlorophyll co-localization in the transfected cell. GFP expression in a single organelle per cell that was larger than a chloroplast and not co-localized to a chloroplast was presumed to be in the nucleus. Additional punctate staining was observed that did not correspond to chloroplasts, but was similar to the expression observed with the *TTL* (peroxisomal-targeted) vector (Figure 4g,h,i) and was attributed to peroxisomal targeting. Typical peroxisomes are labeled with arrows in Figure 4g,i.

Transiently expressed TriTag-2-GFP (Figure 6) display cytosolic localization, as well as a bright punctate pattern indicating a higher level of peroxisomal targeting and a lower signal in the chloroplasts. Overall, TriTag-1 localized GFP preferentially to the chloroplasts, while TriTag-2 localized this protein to the peroxisomes, with similar targeting to the cytoplasm, as evidenced by GFP localization at the cell periphery and presumably the nucleus.

### Subcellular localization of TriTag-3 fused to GFP

**Figure 7:**
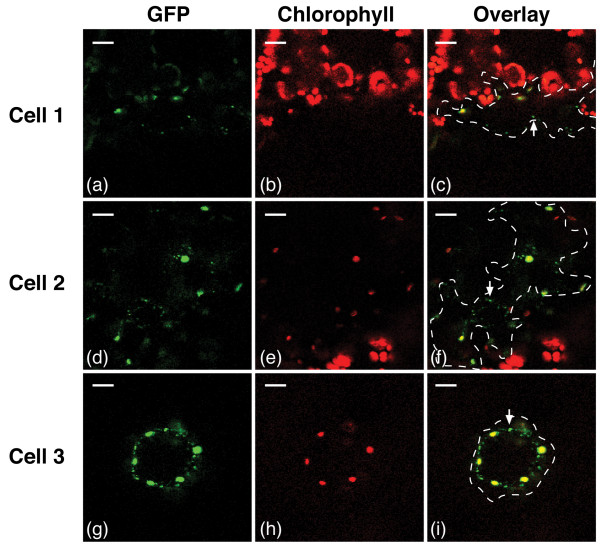
**TriTag-3-GFP distribution in *N. benthamiana *leaf epidermal cells.** Images of GFP expression **(a, d, g)**, chlorophyll autofluorescence **(b, e, h)**, and composite **(c, f, i)** of three different cells were generated by confocal microscopy. Arrows indicate fluorescing peroxisomes. While a clear cellular periphery is not distinguishable in the GFP signal, essentially no fluorescence is observed in the cytosol. Scale bars 25 μm.

TriTag-3 was designed to contain the *TTL* peroxisomal targeting sequence within the PIMT2 chloroplast sequence (Figure 2). *N. benthamiana* epidermal leaf cells transiently expressing TriTag-3-GFP display chloroplast localization and punctate peroxisomal localization (Figure 7). Essentially no GFP was observed in the cytosol. This observation indicates that the hybrid chloroplast/peroxisome targeting sequence is efficiently recognized by the corresponding localization systems, and also that the cytoplasmic plus nuclear localization observed with TriTags 1 and 2 is likely due to mRNAs spliced so that they lack both the peroxisomal and chloroplast targeting sequences.

## Discussion

In this study, we describe simple strategies for localizing a single transgenic protein to multiple cellular compartments in plants. Variation in N-terminal targeting sequences was presumably encoded by alternative splicing of the encoding mRNA or simultaneous function of multiple-targeting sequences as expressed in a single protein. This greatly economizes the amount of DNA transfected. In addition, dual targeting was achieved by an ambiguous N-terminal signal with elements of chloroplast and peroxisomal targeting sequences. We designed three different short, N-terminal elements for coordinate chloroplast, peroxisome and cytosol targeting, termed ‘TriTags”. TriTag-1 and TriTag-2 (Figure 1) were designed by combining naturally occurring DNAs encoding alternatively spliced mRNAs that direct the encoded proteins to either the chloroplast plus cytoplasm [16] or the peroxisome plus cytoplasm [21]. TriTag-3 (Figure 2) does not rely on alternative splicing and consists of a chloroplast targeting sequence in which a naturally unstructured portion has been replaced with a peroxisomal targeting sequence [31].

The TriTags function *in vivo* to target GFP in *Nicotiana benthamiana* leaf epidermal cells (summarized in Figure 3). We compared confocal images of the TriTags to controls of untagged GFP, and GFP with N-terminal tags that had previously been shown to target chloroplasts and peroxisomes [15,16,20,21]. Plasmid DNA was delivered into leaf cells by standard biolistic transfection. Untagged GFP was localized to the cytoplasm and nucleus, with some nuclear localization being expected because the nuclear pore has a large, aqueous channel that permits entry of molecules up to about 70 kD. TriTag-1 and TriTag-2 mediated GFP expression in the chloroplast, peroxisome, and cytoplasm (plus nucleus), with TriTag-1 showing a slight preference for the chloroplast over peroxisome and TriTag-2 showing the opposite behavior. TriTag-3 mediated strong localization to both the peroxisome and chloroplast, but not detectably to the cytoplasm. These behaviors suggest that alternatively spliced forms of TriTag-1 and TriTag-2 are being produced (Figures 1 and 3).

The re-engineering of photorespiration pathways [1] illustrates the potential utility of such multiple-targeting elements. Normally during photorespiration, glycolate is generated in the chloroplast and then transported into the cytoplasm and then into the peroxisome, where it is oxidized to glyoxylate in an O_2_-dependent reaction. The reduction of oxygen, rather than NAD(P)^+^ as an oxidizing agent represents a waste of reducing equivalents and energy. Kebeish et al. [1] engineered plants to express in the chloroplast and NAD^+^-dependent bacterial glycolate metabolizing pathway and found this enhanced the growth of light-limited *Arabidopsis*. In this situation, the added bacterial pathway competes with transport of glycolate from the chloroplast into the cytoplasm. Expression of the pathway in the cytoplasm and peroxisome could further enhance the amount of glycolate that is metabolized by this more efficient pathway. However, as the pathway involves five polypeptide coding sequences, expression of all proteins in three compartments could be prohibitive.

Our results also suggest that novel alternative splicing systems can be engineered in a straightforward manner. One risk in designing such systems is that it is difficult to predict the efficiency of a novel alternatively spliced system. Future designs may use different alternatively-spliced base genes, whose splice ratios may be better known for quantitation. Further experiments with a more stable vector delivery system (e.g. *Agrobacterium* transfection) could give us additional material for quantitative PCR or changes in expression over time, and further inform future designs based on engineered alternative splicing.

## Conclusions

Plant metabolic engineering remains a formidable effort in terms of time and resources. The field requires simple and efficient technologies for transforming plants with multi-functional proteins. Our system with engineered alternative splicing could be used to target a single transgene to multiple locations, namely the chloroplast, cytosol, and peroxisome. In addition, we demonstrated that a peroxisomal signal embedded within a chloroplast signal allows dual targeting of the transgene. These devices may reduce time and resources spent on plant metabolic engineering.

## Methods

### Strains and plasmids

*E. coli* K12 strains (NEB Turbo, New England Biolabs) were used as plasmid hosts for cloning work on binary vectors for transient expression and/or stable genomic integration. Plasmids (Table 1), were constructed with traditional cloning methods [32], BglBricks [33], BioBricks [34], or Gibson assembly [35]. *E. coli* K12 cells were grown in Luria-Bertani medium with appropriate antibiotics (100 μg/mL kanamycin).

**Table 1:** Plasmids constructed in this study

pORE-GFP	pORE binary vector expressing untagged soluble modified GFP (smGFP) controlled by the pENTCUP2 promoter [29].
pORE-PIMT2-GFP	pORE vector expressing *A. thaliana* codon-optimized GFP, with an N-terminal chloroplast targeting peptide of protein-L-isoaspartate methyltransferase (*PIMT2*) [15,16].
pORE-TTL-GFP	pORE vector expressing *A. thaliana* codon-optimized GFP, with an N-terminal start codon and peroxisome-targeting peptide of transthyretin-like S-allantoin synthase (*TTL*) [20,21]
pORE-TriTag-1-GFP	pORE vector expressing TriTag-1-fused GFP controlled by the pENTCUP2 promoter.
pORE-TriTag-2-GFP	pORE vector expressing TriTag-2-fused GFP controlled by the pENTCUP2 promoter.
pORE-TriTag-3-GFP	pORE vector expressing TriTag-3-fused GFP controlled by the pENTCUP2 promoter.

### TriTag synthesis and cloning

TriTag-1, TriTag-2 and TriTag-3 were synthesized (GeneBlocks, IDT, Coralville, IA), and cloned in-frame 5′ of the soluble modified GFP (smGFP) using Gibson assembly [35]. This modified GFP contains three site-directed mutations that increase the protein’s solubility and fluorescence intensity [36]. Based on splice site prediction with NetPlantGene [37], we predicted that the processed spliceforms of TriTag-1 and TriTag 2 encodes for GFP variants containing regions for chloroplast targeting, peroxisomal targeting or neither. Spliceforms other than those found using NetPlantGene would either incorporate a stop codon or lack organelle-targeting information, causing premature translation or sole targeting to the cytosol, respectively.

### Plant material

All plants were incubated at 16-20°C in a 16/8 hour light/dark cycle and watered twice weekly. Peat-based soil-free media (Metromix, SunGro Horticulture, Vancouver, Canada) was autoclaved 45 min before use. Leaves from 3–5 month old *Nicotiana benthamiana* seedlings plants were collected for bombardment.

### Biolistic delivery

DNA-gold particle complexes for biolistic delivery were prepared according to manufacturer’s instructions for use with the Helios Gene Gun (Bio-Rad, Hercules, CA) as follows: Plasmid DNA (50 μg) containing the tagged GFP gene was pelleted onto 1 μm gold particles (6–8 mg) in a spermidine (100 μL, 0.05 M) and CaCl_2_ (100 μL, 1.0 M) mixture and resuspended in a polyvinylpyrrolidone/EtOH solution (5.7 mg/mL). The resulting suspension was deposited onto the inside surface area of Tygon plastic tubing (o.d. = 2 mm) and diced into cartridges facilitated by the Tubing Prep Station (Bio-Rad, Hercules, CA). Cartridges were stable up to 6 months dessicated at 4°C. The underside of *Nicotiana benthamiana* leaves were transformed biolistically using the Helios Gene Gun (Bio-Rad, Hercules, CA) at 150–250 psi He [38]. The leaves were placed on wet filter paper in Petri dishes and stored on a bench-top under ambient lighting and room temperature for 48 hours before imaging analysis.

### Target control proteins

As expected, control proteins showed untagged smGFP was distributed extensively in the cytosol and nucleus (Figure 4a,b,c), but not the vacuole, which makes up the bulk of the plant cell volume. This localization pattern matches previous untagged GFP localization studies [30]. Cytosolic and chloroplast localization controls were determined by transient expression of GFP fused to the *PIMT2* chloroplast targeting peptide and the native autofluorescence of chlorophyll (Figure 4d,e,f). Peroxisomal localizations were determined by comparing images to transient expression of GFP fused to the *TTL* peroxisomal targeting peptide, which matches previous localization studies [21].

### Prediction software

Splice junctions within the TriTag-1 and TriTag-2 sequences were predicted using the NetPlantGene server [37]. Targeting to the chloroplast and peroxisome of the TriTag-1 and TriTag-2 splice variants and TriTag-3 were predicted using TargetP [26] and PeroxisomeDB 2.0 [27]. Peptide structures of CTPb and TriTag-3 were determined using PROFbval on the ROSTLAB server [23].

### Imaging and processing

Bombarded leaves were diced and placed on glass slides in 0.1% Triton-X100 and imaged by fluorescence confocal microscopy (excitation at 489 nm, detection at 500–569 for GFP and 630–700 for chlorophyll autoflourescence) using a 40x water-based objective (numerical aperture 1.10).

## Abbreviations

PTS2: Peroxisome targeting signal 2; TTL: *Arabidopsis* transthyretin-like S-allantoin synthase gene; CTP: Chloroplast targeting peptide; PIMT2: *Arabidopsis* protein-L-isoaspartate methyltransferase gene; CTPa: Chloroplast targeting peptide from *PIMT2*; rbcS1: *Solanum tuberosum* ribulose-1,5-biphosphate carboxylase (RuBisCO) small-subunit gene; CTPb: Chloroplast targeting peptide from *rbc*S1; smGFP: Soluble modified green fluorescent protein.

## Competing interests

PAS, JCW, and MdM have founded in Chimerion Biotechnology, Inc., on related research.

## Authors’ contributions

PAS, JCW, and MdM conceived of the research; MV and MdM designed the study and performed the experiments; MV, JCW and MdM analyzed the data and wrote the manuscript. All authors read and approved the final manuscript.
